# Polysaccharide hydrogel based 3D printed tumor models for chemotherapeutic drug screening

**DOI:** 10.1038/s41598-020-79325-8

**Published:** 2021-01-11

**Authors:** Aragaw Gebeyehu, Sunil Kumar Surapaneni, John Huang, Arindam Mondal, Vivian Ziwen Wang, Nana Fatima Haruna, Arvind Bagde, Peggy Arthur, Shallu Kutlehria, Nil Patel, Arun K. Rishi, Mandip Singh

**Affiliations:** 1 College of Pharmacy and Pharmaceutical Sciences, Florida A&M University, Tallahassee, FL 32307 USA; 2 TheWell Bioscience, North Brunswick, New Jersey 08902 USA; 3 John D. Dingell VA Medical Center, Karmanos Cancer Institute, Department of Oncology, Wayne State University, Detroit, MI 48201 USA

**Keywords:** Cancer microenvironment, Drug discovery, Materials science

## Abstract

A series of stable and ready-to-use bioinks have been developed based on the xeno-free and tunable hydrogel (VitroGel) system. Cell laden scaffold fabrication with optimized polysaccharide-based inks demonstrated that Ink H4 and RGD modified Ink H4-RGD had excellent rheological properties. Both bioinks were printable with 25–40 kPa extrusion pressure, showed 90% cell viability, shear-thinning and rapid shear recovery properties making them feasible for extrusion bioprinting without UV curing or temperature adjustment. Ink H4-RGD showed printability between 20 and 37 °C and the scaffolds remained stable for 15 days at temperature of 37 °C. 3D printed non-small-cell lung cancer (NSCLC) patient derived xenograft cells (PDCs) showed rapid spheroid growth of size around 500 µm in diameter and tumor microenvironment formation within 7 days. IC_50_ values demonstrated higher resistance of 3D spheroids to docetaxel (DTX), doxorubicin (DOX) and erlotinib compared to 2D monolayers of NSCLC-PDX, wild type triple negative breast cancer (MDA-MB-231 WT) and lung adenocarcinoma (HCC-827) cells. Results of flow property, shape fidelity, scaffold stability and biocompatibility of H4-RGD suggest that this hydrogel could be considered for 3D cell bioprinting and also for in-vitro tumor microenvironment development for high throughput screening of various anti-cancer drugs.

## Introduction

Traditionally, anticancer drugs are evaluated in conventional two-dimensional (2D) cell culture platforms. However, conventional 2D cultured cancer cells cannot mimic the complexity and heterogeneity of in-vivo tumors, which usually grow in a three-dimensional (3D) conformation^1–4^. To overcome this, various 3D cell culture platforms are being currently developed, which can mimic the in-vivo tumor microenvironment as spheroid cells or organoid models. Recent 3D cell culture models narrow the gap between 2D cell culture models and animal disease models by mimicking/recapitulating in-vivo or natural 3D tumor microenvironment. They are also useful in drug discovery for determining the sensitivity of chemotherapeutics to tumor cells both at the cellular and molecular level^2,5,6^.

Hydrogel based 3D scaffolds are particularly gaining attention due to their cell encapsulation capability and property of mimicking native extracellular matrix (ECM)^7,8^. Biochemical, mechanical and physical properties of hydrogels determine 3D tumor microenvironment construction in-vitro. Under patho-physiological conditions, tumor cells have the tendency to bisect stromal and connective tissues depending on the ECM stiffness, porosity and linearity. Ideal bioprinted cell-laden scaffolds majorly mimic the stiffness of in-vivo tumor microenvironment^9–11^.

Rheological modifications of the hydrogel can directly influence bioprinting, scaffold shape fidelity, viability and proliferation of cells^12–14^. These also aid in mimicking in-vivo tumor stiffness and native ECM viscoelastic properties. Flow property and tensile strength are the two rheological parameters for bioink extrusion and scaffold formation^12,13,15^. Collagen, fibrin, gelatin, alginate and hyaluronic acid are the widely used natural polymeric biomaterials for bioink formulation because of their resemblance to natural extracellular matrix (ECM) components^16–18^. VitroGel is a ready-to-use, xeno-free hydrogel system for 3D cell culture research. It can be modified with multi-functional ligands and mechanical strength to closely mimic the natural extracellular matrix (ECM) environment to support a wide range of cell types for different applications. The hydrogel is room temperature stable, has a neutral pH, transparent, permeable and compatible with different imaging systems. The solution transforms into a hydrogel matrix by simply mixing with the cell culture medium.

Biocompatibility, hydrophilicity, biomimetic ability, bio-processability, biodegradability, and affordability of the bioinks are also taken into consideration during 3D printing of cell laden scaffolds^16^. During 3D printing, less viscous bioinks easily flow through the narrow printing nozzle but they have the drawbacks of smearing and low shape fidelity^12,19^. Highly viscous bioinks can give better shape resolution by using high extrusion pressure^16,19^ but creation of high shear force in the printing nozzle by application of higher extrusion pressure causes cell destruction during printing process^16,20^. So, optimizing the flow properties is the primary criteria of printable bioinks^21–23^.

Polymer crosslinking method is also another key parameter of the bioink for 3D printed scaffolds fabrication^24–26^. Crosslinking by hydrogen bonds, crystallization, ionic interactions and protein interaction are examples of physical crosslinking mechanisms^26,27^. Physically crosslinked bioinks are usually associated with poor mechanical stability. Covalent bonding between polymer and crosslinker functional groups is an example of chemical crosslinking mechanisms^28^. Among various methods, covalent crosslinking is proved to be an effective method for improving the physiological stability of printed structures^29,30^. However, toxicity and unwanted reactions of the crosslinker with the bioactive substances present in the hydrogel matrix limit their application. Changes in pH and temperature, high energy radiation, free radical polymerizations can be used for crosslinking in 3D cultures^27^.

In this study, we hypothesize to test a series of room temperature stable and ready-to-use hydrogels by working in collaboration with TheWell Bioscience (North Brunswick, NJ 08902) company. Our goal is to develop a hydrogel which mimics the in-vivo tumor microenvironment, shows good printability, rheological characteristics (i.e., shear-thinning and rapid recovery rheological properties of hydrogels can lead to a stable printed structure after extrusion), biocompatibility and also can be used for application of screening of anti-cancer drugs. As current crosslinking methods of bioinks such as sudden changes in temperature, chemical reactions, and exposure to high energy radiation (UV light) could cause destruction to the cells during the process of solidification of printed scaffold after hydrogel extrusion^31,32^, we would also like to have our printed cell laden scaffolds developed with characterized hydrogel have additional property of getting crosslinked with the cell culture media, maintaining good biocompatibility and stability for long periods of time. Since there are no requirements of crosslinking through temperature changes, chemicals and radiation during bioprinting, this novel bioink could be an easy-to-use system to carry out comparative cytotoxicity testing of various anticancer drugs in 3D printed spheroids and conventional 2D cultures.

## Results

### Bioink optimization for scaffold fabrication and bioink cytocompatibility studies

**Figure 1:**
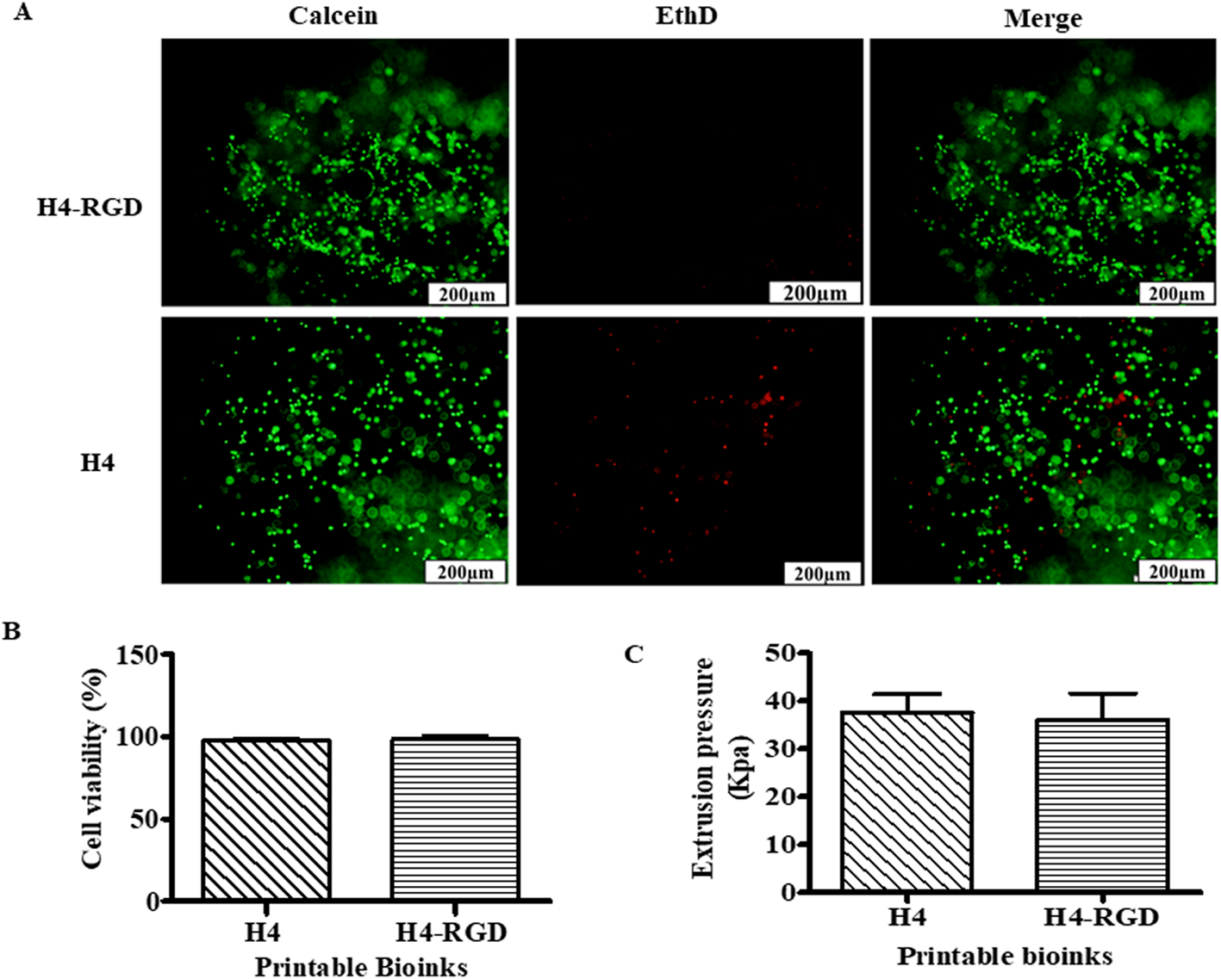
Ink-H4-RGD showing good extrudability and biocompatibility in MDA-MB-231 WT cells. (**A**) Live/dead cell assay analysis using Calcein AM/Ethidium homodimer III staining. Results shown were representative of three independent experiments. Microscopic images were acquired and analysed by using Olympus Microscope IX71 and cellSens Standard software (version 1.16; Tokyo, Japan) respectively. (**B**) Post-printing cell viability of MDA-MB-231 WT cells, which is expressed as percent viability as analysed by NIH ImageJ software (1.43u; https://imagej.nih.gov/ij). (**C**) Extrusion pressure required for printing of bioinks.

**Figure 2:**
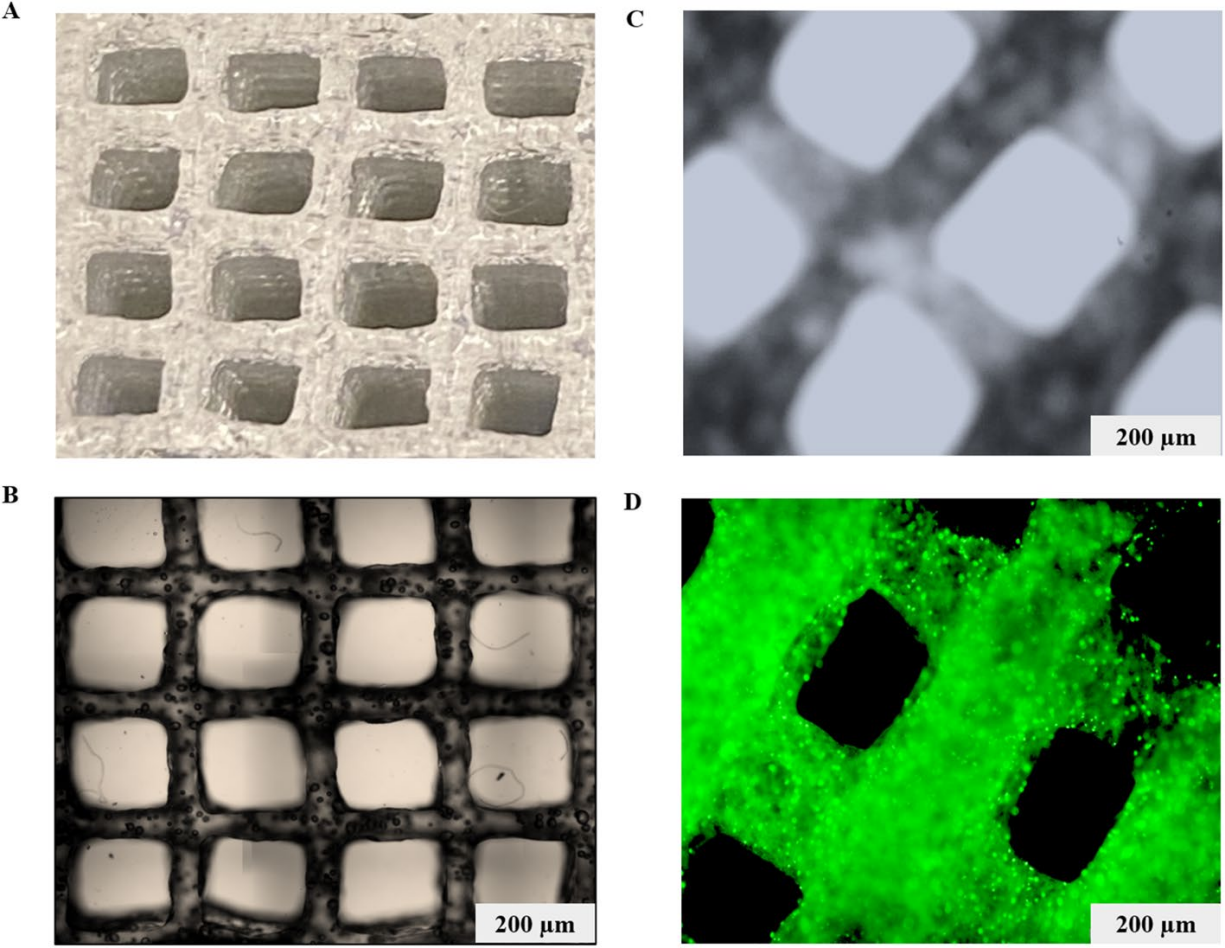
Extrusion uniformity and shape fidelity of printed scaffold. (**A**) Photographic image of ten layer printed scaffold with Ink H4-RGD. (**B**, **C**) Microscopic images showing the pore sizes and line width of printed scaffolds with Ink H4-RGD; (**D**) Microscopic images showing the cell (NSCLC-PDX) laden Ink H4RGD scaffold after staining with Calcein. Microscopic images were acquired by using Olympus Microscope IX71 and cellSens Standard software (version 1.16; Tokyo, Japan).

**Figure 3:**
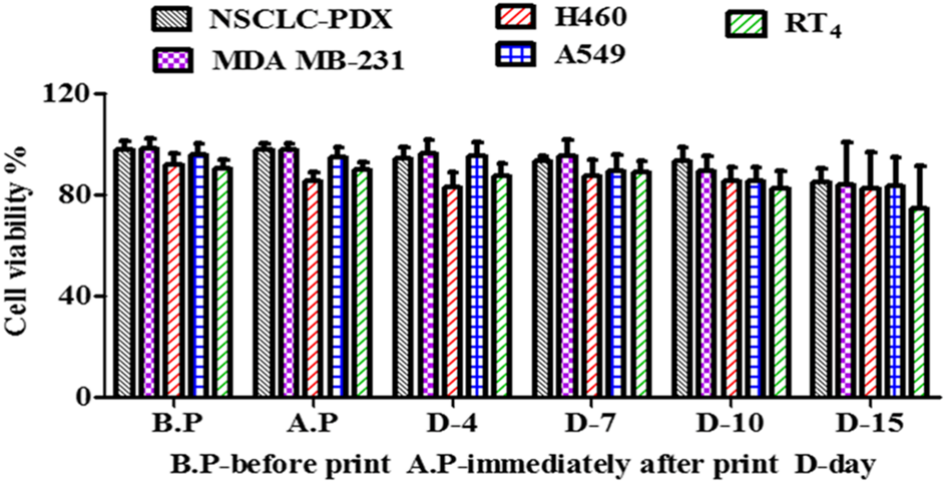
Biocompatibility of Ink H4-RGD. Ink H4-RGD biocompatibility in NSCLC-PDX, RT4, MDA-MB-231, H460 and A549 cell lines before printing, immediately after printing, on Day 4, Day 7, Day 10 and Day 15 by counting cell viability through NIH ImageJ software (1.43u; https://imagej.nih.gov/ij). Results shown were representative of three independent experiments.

After testing a series of room temperature stable and ready-to-use hydrogels received from TheWell Bioscience (North Brunswick, NJ 08902), we found that Ink H4 and its modified form, H4-RGD showed high shape fidelity along with high cell viability. Both the hydrogels showed good viability of MDA-MB-231 WT cells after immediate post-printing evaluation by performing Calcein AM/Ethidium homodimer III staining, which showed the images of live (stained green) and dead (stained red) cells (Fig. 1A) and the percentage of cell viability (Fig. 1B) was calculated by NIH ImageJ software. We observed that both these inks were printed under pressure conditions ranging from 25 to 50 kPa (Fig. 1C). We observed poor printability with Ink H1 (Supplementary information; Fig. S1A) but we have seen good viability in printed Ink H1 cell laden scaffolds (Supplementary information; Fig. S2). Ink H2 showed printability (Supplementary information; Fig. S1B) and good cell viability (Supplementary information; Fig. S2) but the printed scaffolds were not stable for further assays. Although the printability was good with Ink H3 (Supplementary information; Fig. S1C), it used high extrusion pressure (i.e., approximately 180 kPa) for printing (Supplementary information; Fig. S3). Ink H3 printed scaffolds showed poor cell viability (Supplementary information; Fig. S2). It was observed that extrusion pressure of approximately 60 kPa was used for printing of Ink H5 cell laden scaffolds (Supplementary information; Fig. S3). Ink H5 printed cell laden scaffolds showed 85% viability (Supplementary information; Fig. S2). Figure 2A shows the photographic image of 10-layer printed scaffold with Ink H4-RGD. The printed scaffolds of Ink H4-RGD displayed designed pore size and good line width without any smearing problems (Fig. 2B,C). Figure 2D shows the fluorescent image of NSCLC-PDX cell laden Ink H4-RGD scaffold stained with calcein. The average Pr (printability) value of 12 Ink H4-RGD scaffolds was 0.96, which indicates that this ink could be considered for 3D printing of cell laden scaffolds. We also investigated the biocompatibility of Ink H4-RGD in various cancer cells and found that all evaluated cell lines of lung cancer (NSCLC-PDX, H460, HCC-827and A549) and breast cancer (MDA-MB-231WT), and bladder cancer (RT4) cells have showed more than 90% cell viability after post printing evaluation as analysed by counting the cell viability through NIH ImageJ software (Fig. 3).

### Rheological properties of optimized bioprintable inks

**Figure 4:**
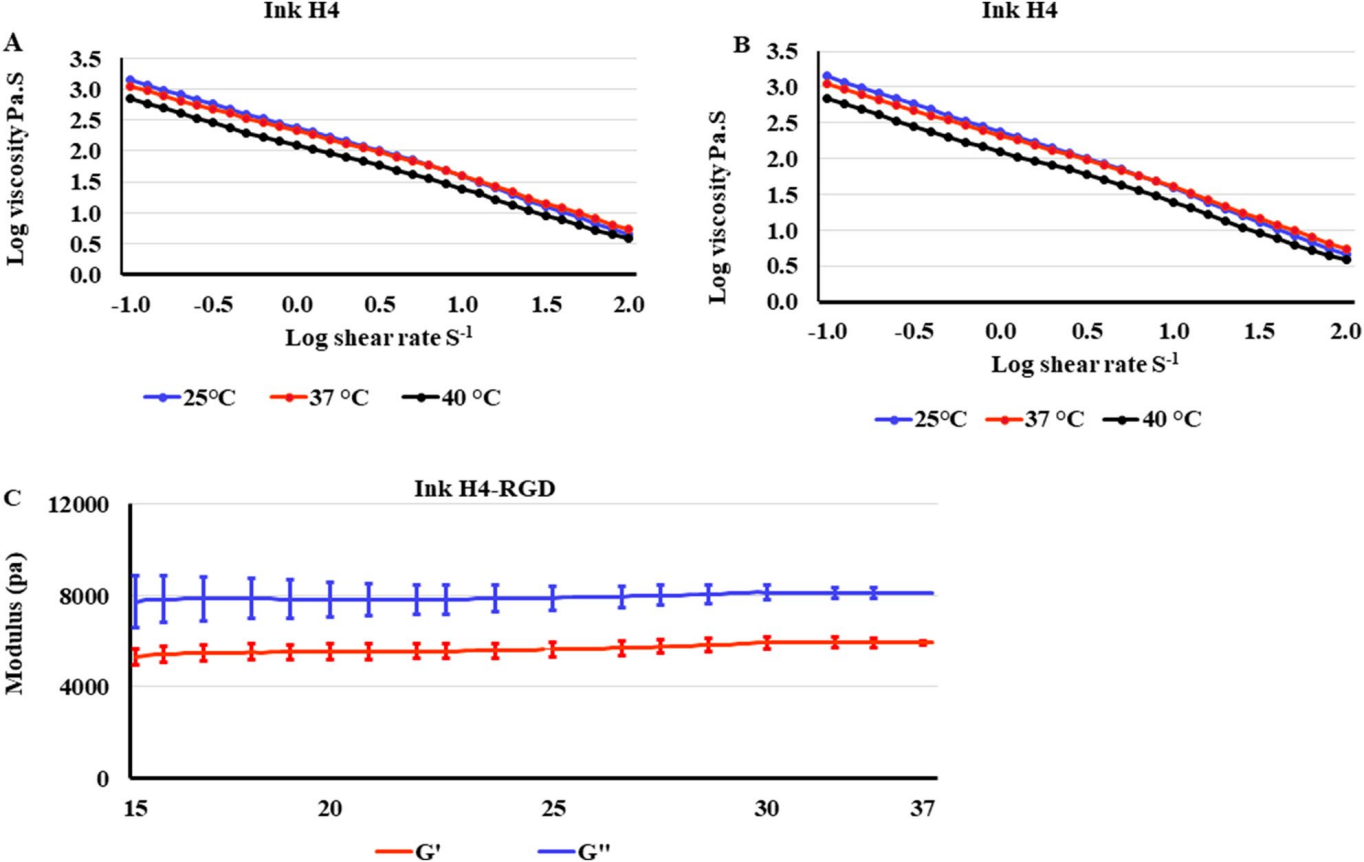
Elastic modulus and viscosity of Ink H4 and Ink H4-RGD under temperature and time sweep test. (**A**) The viscosity of Ink H4 with different shear rates at 25 °C, 37 °C and 40 °C. (**B**) The viscosity of Ink H4-RGD with different shear rates at 25 °C, 37 °C and 40 °C. (**C**) Viscoelasticity (modulus) of Ink H4-RGD at different temperatures.

**Figure 5:**
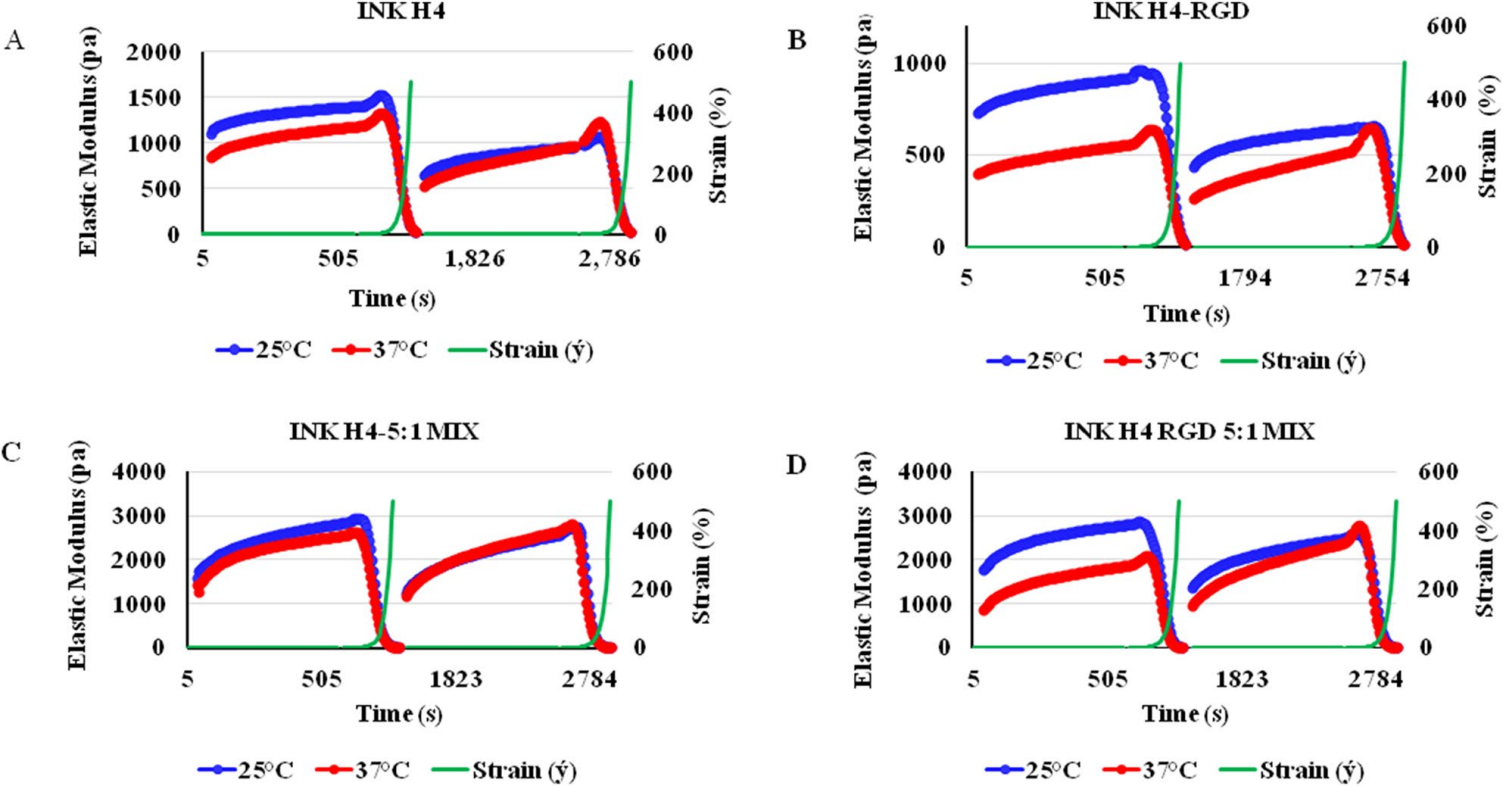
Shear-thinning and rapid shear recovery properties of Ink H4 and Ink H4-RGD by oscillation-amplitude sweep test. Two sets of the oscillatory amplitude sweep tests were performed for each bioink (0.1–500% shear strain, 10 min each). Oscillation strain above the critical strain disrupts the network of the hydrogel, indicating shear thinning property. The initial G′ of the second test indicated the rapid healing of the mechanical property after the first test. (**A**) Repeated shear-thinning and shear recovery properties of Ink H4 at both 25 °C and 37 °C; (**B**) Repeated shear-thinning and shear recovery properties of Ink H4-RGD at both 25 °C and 37 °C; (**C**) Repeated shear-thinning and shear recovery properties of Ink H4 5:1 mix (cross-linked) with cell culture media at both 25 °C and 37 °C; (**D**) Repeated shear-thinning and shear recovery properties of Ink H4-RGD 5:1 mix (cross-linked) with cell culture media at both 25 °C and 37 °C.

We evaluated rheological parameters to assess and determine the feasibility of inks H4 and H4-RGD for extrusion based bioprinting without additional curing process. Rheological assessments revealed that Ink H4 and Ink H4-RGD displayed shear thinning properties i.e., we observed decrease in the viscosity when the shear rate was increased (Fig. 4A,B) at 25 °C. This suggests that inks H4 and H4-RGD can be used for extrusion based bioprinting. Further, oscillation temperature sweep test demonstrated that Ink H4-RGD displayed stable elastic modulus across wide temperature ranges i.e., at room temperature and physiological temperature (Fig. 4C). Further, we observed that Inks H4 and H4-RGD showed shear thinning and shear recovery properties at both 25 °C and 37 °C (Fig. 5A,B). Further, we assessed the rheological properties after crosslinking of Ink H4 and H4-RGD with cell culture media in 5:1 ratio. It was observed that crosslinked inks H4 and H4-RGD also showed shear thinning and shear recovery properties at both 25 °C and 37 °C (Fig. 5C,D). However, the recovery rate was faster at 37 °C in comparison to 25 °C, and also faster with 5:1 mixed (partially crosslinked) samples when compared to unmixed samples (Fig. 5A–D). We observed that all the four samples recovered more than 60% of the elastic modulus instantly after 500% shear strain force was ceased, and almost 100% of the elastic modulus during the 10 min time sweep test, indicating the shear recovery properties of the hydrogel. After the phase 3 test, phase 4 amplitude sweep test was applied to the same samples and we assessed rheological properties. We again observed similar shear-thinning properties of the hydrogel system, further confirming the above results. Since the mechanical strength of the inks can be rapidly recovered after the shearing force was ceased, these inks can maintain the printing structure without further curing process. Inks H4 and H4-RGD have showed pseudoplastic or shear thinning behaviour. The plot of log shear stress vs log shear rate has also showed non-zero intercept. Based on the power law mode equation, we have summarized the flow index (n), flow consistency index (k) and the R^2^ values of Ink H4 and H4-RGD at 25 °C, 37 °C and 40 °C in Table 1. The n value was found to be less than 1, which also indicates non-Newtonian pseudoplastic flow properties of Inks H4 and H4-RGD.

**Table 1:** Flow index and consistency index of Inks H4 and Ink H4-RGD at 25 °C, 37 °C and 40 °C.

Hydrogel	INK-H4			INK-H4 RGD		
Temperature	25 °C	37 °C	40 °C	25 °C	37 °C	40 °C
n	0.1252	0.1925	0.2192	0.1251	0.2101	0.2634
k	269.5	237.7	137.1	277.4	178.6	102.2
R^2^	0.8929	0.9511	0.969	0.9683	0.9911	0.9922

### Mechanical properties of printable hydrogel and stiffness of cell-laden scaffolds

**Figure 6:**
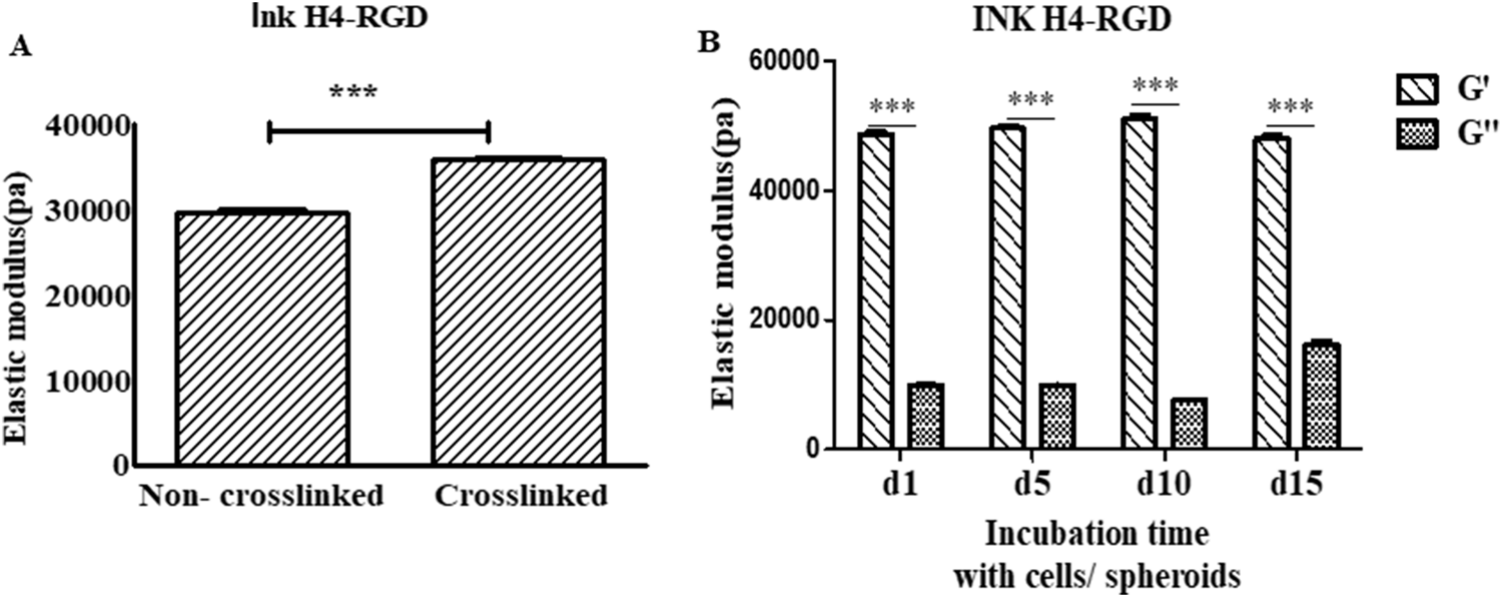
Rheological properties of the hydrogel and stiffness of cell laden scaffolds. (**A**) Elastic modulus of crosslinked Ink H4-RGD without cells/spheroids. (**B**) Elastic modulus of cell laden scaffolds when incubated at 37 °C and 5% CO_2_ for 15 days.

Based on our printability, cell viability and rheological parameters experimental results, Ink H4-RGD was optimized for 3D printing of cancer cells in-vitro. It was hypothesized that this hydrogel would offer high printability and compressive modulus. Both non crosslinked H4 RGD and partially crosslinked H4-RGD (5:1 of hydrogel and cell culture media) displayed elastic modulus of around 30 kPa after 24 h incubation with excess cell culture medium at 37 °C (Fig. 6A). Further, the stiffness of printed NSCLC-PDX cell laden scaffolds was also evaluated by using oscillation-time sweep measurements on day 1, 5, 10 and 15 (Fig. 6B). The rheological data of cell laden scaffolds showed around 50 kPa elastic modulus and 7 kPa loss modulus during 15 days of incubation. The loss factor was calculated by dividing the average loss modulus with elastic modulus (G″/G′). The loss factor of printed cell laden scaffolds was found to be in between 0.14 and 0.4 during 15 days of incubation period (Fig. 6B).

### Characterization of 3D spheroids and assessment of *in-vitro* tumor microenvironment, which mimics the in vivo stromal characteristics

**Figure 7:**
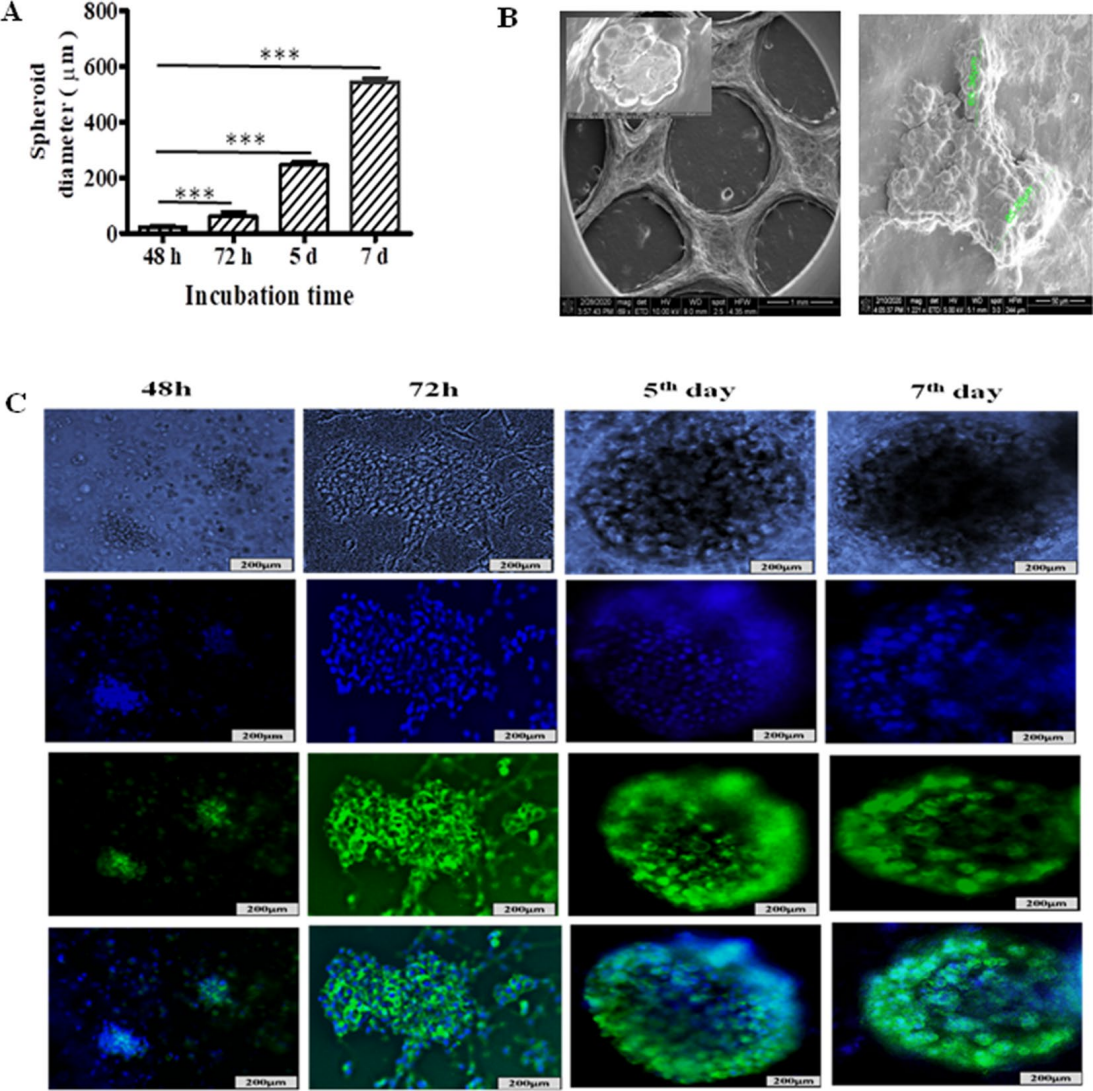
3D printed NSCLC-PDX cells/spheroids growth in printed ink H4-RGD scaffolds. (**A**) Average spheroid proliferation/growth rate over a period of 7 days. (**B**) SEM images of spheroids inside the ink H4RGD scaffold. (**C**) Spheroid images captured in bright field microscopy and also after staining with NucBlue/Actin Green staining after 48 h, 72 h, on 5th day and 7th day. Microscopic images were acquired by using Olympus Microscope IX71 and cellSens Standard software (version 1.16; Tokyo, Japan).

**Figure 8:**
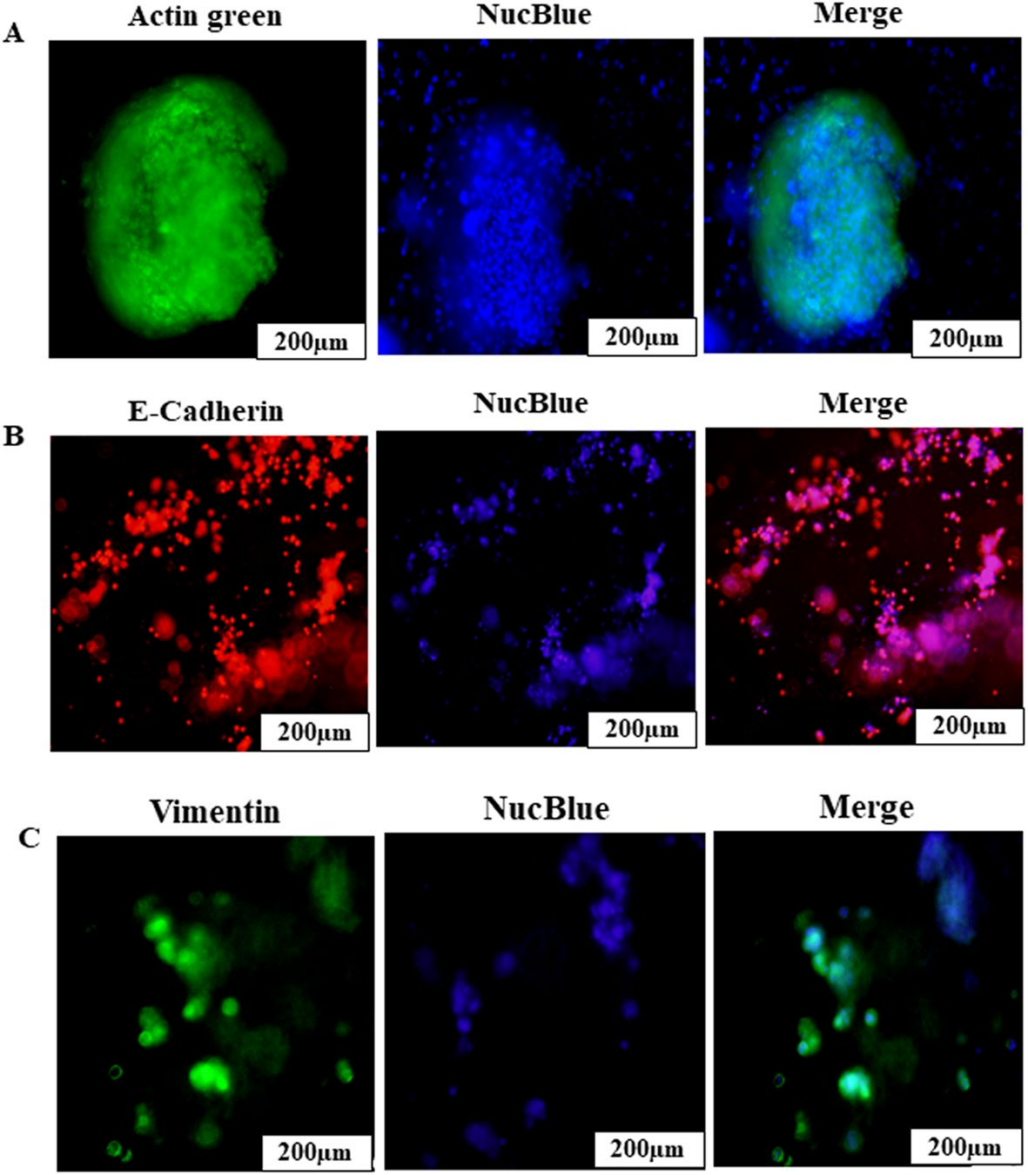
3D spheroids formation in printed ink H4-RGD NSCLC-PDX cell laden scaffolds. (**A**) Spheroid formation in NSCLC-PDX cells with ink H4-RGD on the 7th day after staining with NucBlue/Actin Green staining. (**B**) Immunofluorescence staining for E-cadherin in printed NSCLC-PDX laden scaffold with ink H4-RGD; (**C**) Immunofluorescence staining for Vimentin in printed NSCLC-PDX laden scaffold with ink H4-RGD. Microscopic images were acquired by using Olympus Microscope IX71 and cellSens Standard software (version 1.16; Tokyo, Japan).

NSCLC-PDX cells were grown in printed scaffolds for 7 days during which the size of spheroids was measured with Olympus IX71 microscope and cellSens Standard software (version 1.16; Tokyo, Japan) after printing on 48 h, 72 h, 5th day and 7th day. The spheroidal growth and the diameter of spheroids was found to be in the range of 100–500 µm during the first 7 days (Fig. 7A). We also characterised 3D spheroids by using SEM, which showed the presence of spheroids inside the printed scaffold of Ink H4-RGD (Fig. 7B). We checked the growth of 3D NSCLC-PDX spheroids by performing NucBlue/Actin green staining for a period of 7 days (Fig. 7C). Figure 8A depicts the image of 3D NSCLC-PDX spheroids with Ink H4-RGD on day 7 after assessed by using NucBlue/Actin green staining. Further, we assessed the presence of tumor microenvironment by performing immunofluorescence assay for E-cadherin and vimentin, which revealed the expression of cell adhesion molecule, E-cadherin and Vimentin in the printed NSCLC-PDX 3D spheroids (Fig. 8B,C).

### Comparative 3D and 2D culture platform anticancer drug sensitivity evaluation

We investigated the anti-cancer efficacy of DTX, erlotinib and DOX in the printed 3D cell laden scaffolds formed by using Ink H4-RGD hydrogel and compared their efficacy in 2D monolayers. In all the three tested cell lines (MDA-MB-231WT, NSCLC-PDX and HCC-827), 3D spheroids showed more resistance to DTX, erlotinib and DOX in comparison to 2D monolayers. The IC_50_ values for DTX in 2D monolayers of NSCLC PDX, MDA-MB-231WT and HCC827 cells were found to be 1.63, 1.82 and 6.83 µM, respectively and the IC_50_ values in 3D printed spheroids (NSCLC -PDX, MDA-MB-231WT and HCC-827 cells) were found to be 16.2, 15.1 and 64.5 µM respectively (Table 2). The IC_50_ values of erlotinib in 2D monolayer cells of NSCLC-PDX, MDA-MB-231WT and HCC-827 were found to be 52.12, 52.2 and 48.53 µM, respectively. The IC_50_ values of erlotinib in 3D printed spheroids of NSCLCPDX, MDA-MB-231WT and HCC-827 cells were found to be 152.12, 131.53 and 138.79 µM respectively (Table 2). The IC_50_ values of DOX in 2D monolayer cells of MDA-MB 231WT and HCC827 were found to be 1.58 and 10.73 µM respectively (Table 2). The IC_50_ values of DOX in 3D printed spheroids of MDA-MB-231WT and HCC-827 cells were found to be 77.35 and 85.35 µM, respectively. The IC_50_ values in 3D printed cell spheroids ranged from 3 to 90 folds higher than the 2D counterparts with equivalent cell numbers, all these suggesting that tumor complexity contribute to decreased sensitivity of DTX, erlotinib and DOX in 3D spheroids.

**Table 2:** Cytotoxicity of DTX, DOX and Erlotinib in 2D monolayers and 3D spheroids of NSCLC-PDX, MDAMB-231 WT, and HCC-827 cells.

Cell lines	DTX IC_50_ (µM)		DOX IC_50_ (µM)		Erlotinib IC_50_ (µM)	
	2D culture	3D culture	2D culture	3D culture	2D culture	3D culture
NSCLC-PDX	1.63 ± 0.23	16.20 ± 1.67			52.12 ± 3.22	152.5 ± 5.67
MDA-MB-231 WT	1.82 ± 0.32	15.10 ± 2.36	1.58 ± 0.32	77.35 ± 2.36	52.50 ± 5.26	131.53 ± 2.36
HCC827	6.83 ± 0.89	64.50 ± 4.56	10.73 ± 1.65	85.35 ± 6.56	48.53 ± 3.65	138.79 ± 4.56

DOX was not used for NSCLC PDX cells due to its lack of effectiveness against lung cancer. The experimental results were expressed as mean ± standard deviation.

## Discussion

Bioprinting techniques are gaining substantial interest in the field of tissue engineering due to their ability to create complex structures. 3D printing of cell laden scaffolds facilitates homogenous cell/spheroids distribution, homogenous drug diffusion through the entire scaffolds and also opens up new avenues for the fabrication of microtissues^33^. Bioprinting of cells with a designed structure closely mimic in-vivo tissue microenvironment during scaffold-based tissue development^34^. Layer by layer printed scaffolds showed higher cell proliferation and growth when compared to conventional non-printed 3D scaffold cultures^35^. Facilitated media diffusion, easy waste removal and relatively homogenous drug diffusion can be achieved by using 3D bioprinting of cell laden scaffolds with designed pore size and good rheological properties when compared to non-printed 3D cultures^36–39^. Bioprinting can also prevent core necrosis which is the common problem of non-printed polymer-based 3D models^40^. 3D printing has been recently used to develop in-vitro cancer models for chemotherapeutic drug screening^41^. Because of their similarity with the initial avascular growth stage of malignancies, micro-metastases and intra-capillary tumor microregions, 3D spheres of tumor tissue have also been recently explored to investigate and demonstrate the efficacy of various chemotherapeutics^42^.

The imperative step in 3D bioprinting is the formulation of a bioink, which mimics tissue specific intercellular microenvironment^43,44^. In this study, a new formulation of VitroGel RGD-PLUS hydrogel system was used for the first time as a printable bioink for tumor tissue fabrication and in-vitro screening of anticancer drugs. It is an animal origin-free polysaccharide hydrogel system, which is modified with RGD peptide for better cell adhesion. As polysaccharide based bioinks do not have cell attachment motif, they are modified with a tripeptide, Arg-Gly-Asp (RGD) to improve the adhesion properties^45–47^. During screening of printable bioinks, biocompatibility with cells and medium should be determined before the selection of an ideal bioink^48–50^. This is the first study reporting the use of a carbohydrate based bioink, H4RGD for 3D printing of tumor cells and for screening them against anticancer drugs. Mechanism behind the cross-linking involves the interaction of hydrogel molecules with Ca^2+^ or Na^+^ from the cell culture medium. This process is slow and forms a soft hydrogel initially. Adding additional cell culture media on top of the hydrogel would allow more ionic molecules to penetrate into the hydrogel matrix, which further saturates the hydrogel cross linking and forms a solid hydrogel.

Bioink extrudability, printed structure line width and scaffold pore size are the determining factors for bioinks evaluation in extrusion-based printings^51,52^. Moreover, bioinks having shear thinning properties can easily pass through the micro-sized nozzle and avoid cell destructive shear forces at the printing nozzle^26,53–55^. Highly viscous bioinks could give better shape resolution but they induce clogging problems at the nozzle tip and can decrease the viability of cells due to the application of high extrusion pressure^18,19,26,53,56–59^. Low viscous bioinks induce smearing and shape demolition problems^26^. In our study, both Ink H4 and Ink H4-RGD have showed shear thinning and shear recovery properties at ambient temperatures between 25 and 37 °C. Moreover, the recovery-time point of both hydrogels to their original elastic modulus (G′) was observed to be less than one minute after shearing. The shear thinning properties of these inks helped us to print cells with minimum printing pressure, which could also be beneficial for maintaining cell viability. Since the mechanical strength of the ink can be rapidly recovered after the shearing force was ceased, these inks can maintain the printing structure without further curing process. On the other hand, minimum shear duration or rapid recovery properties of the hydrogel are helpful for optimum shape fidelity during scaffold construction^60,61^. During printing process, a solid physical appearance of hydrogel has to be broken or disrupted by minimum shear stress or printing pressure so as to facilitate easy bioink flow through a narrow nozzle without any jamming problem and also to decrease printing related cellular stress^19,60^.

Due to its rapid shear recovering property, our vitroGel hydrogel system can maintain the printing structure without any smearing problems through immediate curing process. However, optimal stiffness and stability of printed cell laden scaffolds, depend upon the cross-linking methods and elasticity nature of the bioinks^36,62^. Crosslinker concentration and crosslinking time determine the viscosity of the hydrogel system^63,64^. Bioinks of 3D scaffold constructs should have a superior crosslinking method which can maintain good tensile strength of the hydrogels and viability of the cells^65^. Among various methods, covalent crosslinking is proved to be an effective method for improving the physiological stability of printed structures^29,30^. Addition of cell suspension into the bioinks often decreases the viscosity and complicates the evaluation of printability^51^. Kajsa et.al demonstrated the usage of nanocellulose-alginate bioink and 90 mM CaCl_2_ (crosslinker) for cartilage tissue engineering^66^. In our study, usage of Ink H4-RGD bioinks demonstrates an advantage of crosslinking with cell culture medium. Due to this crosslinking method, the printed cell laden scaffolds were maintained in good tensile strength throughout the incubation period.

In our study, all the rheological data was recorded with relevant parameters such as shear rate, shear stress, shear strain torque, normal forces, and angular frequencies. The viscosity and visco-elasticity of bioinks were evaluated before printing. Further, stiffness and structural integrity of cell laden scaffolds were also determined. Ink H4-RGD displayed less degradability and excellent tensile strength for an extended period of time among all the tested bioinks, suggesting it as a suitable bioink. Inks H4 and H4-RGD showed favourable flow properties when extruded at the printing pressure ranging from 25 to 50 kPa. The loss factor (tan δ) of Ink H4 and H4-RGD showed suitable viscoelasticity for scaffold fabrication. Our results are supported by an earlier study carried out by Katja et al., which showed that bioinks with viscosity range of 30 mPa–6 × 10^7^ mPa are printable by extrusion type printer^26^.

Stiffness of the printed cell laden scaffolds plays a crucial role in structural integrity, cell proliferation and tumor spheroid growth^67^. Meiyu et al. showed that stiffer (48–53 kpa) substrates facilitated the proliferation of BMMSCs when compared to soft substrates^68^. Morphological changes of the cells are observed when encapsulated in hydrogels of different stiffness. Stiffer substrates generally promote cell spreading, whereas soft substrates induce more spherical cell shape^69^. Cavo et al. demonstrated the impact of stiffness of hydrogel on spheroids size and growth^70^. In our study, the stiffness (i.e., 30–55 kPa) of cell laden scaffolds demonstrate higher spheroidal growth and proliferation. Moreover, the stiffness of the scaffolds did not alter/change throughout the incubation period. Printed scaffolds resolution also depends on printing speed, temperature and nozzle diameter^58,71^. Printing with smaller nozzle diameters gives high shape resolution, but it requires high extrusion pressure, which can cause cellular damage^71^. In our study, ink H4-RGD was printed with different nozzle diameters ranging from 200 to 410 µm and higher cell viability was observed at a diameter of 410 µm.

Lattice design plays an important role in 3D printing of cell laden scaffold constructs, as it helps in mimicking cellular microenvironment^48,72–75^. In 3D printed cell culture models, interconnected porous structure helps for the diffusion of nutrients and waste products^75^. In layer by layer printing, scaffold size and the pore size are the determining factors for their stability^76^. Yong et al. demonstrated that diffusion is superior in 3D grid pattern when compared to non-porous edges^77^. In our study, the designed scaffolds had three layered grid structure with 10 × 10 × 1 dimension, average line width (400 µm) and average inter-scaffold porosity (16 × 10^4^ µm^2^) respectively. This design was favourable for high proliferation of the cells and better nutrients diffusion, which was evident from rapid spheroid formation within seven days of incubation.

3D spheroid cultures can mimic the oxygen and nutrient gradients of in-vivo tumors^78^. Previous anticancer drug studies revealed that 3D spheroids show more chemoresistance in comparison to 2D cell monolayers^79–81^. Tumor heterogeneity is responsible for superior drug resistance in 3D spheroids when compared to 2D culture models, which are devoid of essential tumor microenvironment^82,83^. Poor penetration of drugs through the scaffolds and spheroidal microenvironment barriers are also responsible for superior drug resistance in 3D spheroids^84–86^. Thus, there is a need to develop drugs with better penetrating ability to the most interior sections of tumors. The 3D matrix of hydrogel-based scaffolds closely mimic the physical barriers of ECM present around in-vivo tumors. Decreased diffusion of anti-cancer drugs through the spheroids and hypoxia could also contribute to drug resistance in 3D cultures^87^. Karlsson et al. demonstrated that 3D spheroids were more resistant than 2D monolayer colon cancer (HCT-116) cells to anticancer drugs^88^. Paclitaxel, an anti-proliferative drug, which normally acts on dividing cells showed drug resistance in 3D spheroids due to its reduced effect in the interior spheroidal region, which consists of quiescent cells^89^. Besides, the dynamic intercellular and cell-ECM interactions have a major role in drug resistance in 3D spheroids^3^.

In our study, we have seeded equivalent number of cells i.e., 5 × 10^4^ cells/scaffold/well and evaluated cytotoxicity with DTX, DOX and erlotinib in 2D and 3D bioprinted cultures of NSCLC-PDX, MDA MB 231 WT and HCC-827 cells. IC50 values were significantly higher in 3D spheroids treated with DTX, erlotinib and DOX when compared to 2D monolayers of NSCLC-PDX, MDA-MB-231WT and HCC-B27 cells. These results support that 3D spheroids show more chemoresistance than conventional 2D platforms to DTX, erlotinib and DOX due to the tumor heterogeneity of 3D spheroids. Our results corroborate with previous reports which show that 3D cultures of alginate based scaffolds are highly resistant to DTX when compared to 2D monolayers of both A549 and H460 cells i.e., IC_50_ values of DTX in 3D and 2D monolayers of A549 cells are 118.11 µM and 1.94 µM, respectively and IC_50_ values of DTX in 3D and 2D monolayers of H460 cells are 76.27 µM and 1.41 µM, respectively^91^. DOX showed higher resistance in 3D cultures of A549 cells when compared to 2D monolayers i.e., IC_50_ values of DOX in 3D and 2D monolayers of A549 cells are 10.1 µM and 1.5 µM respectively^92^. A study by Mishra et al., has shown that IC_50_ values of cyclosaplin and DOX are 5.3 and 5.9-fold higher in 3D cultures when compared to 2D monolayers of MDA-MB-231 cells^93^. Lovitt et al., has also demonstrated increased IC_50_ value of DOX from 87 nM in 2D monolayers to 636 nM in 3D cultures of MDA-MB-231 cells^90^. Pickl et al. demonstrated that formation of HER2 homodimers in 3D cultures is responsible for increased cytotoxic effects with trastuzumab in HER2 positive breast and ovarian cancer cells when compared to trastuzumab effect in 2D cultures^94^. However, we observed that 3D spheroids formed by ink H4-RGD for MDA-MB-231 WT, NSCLC-PDX and HCC-827 cells showed more resistance in comparison to 2D cell monolayers. This possibly suggests the role of tumor microenvironment created by Ink H4-RGD in determining the variability of chemotherapeutics response in 3D spheroids. Decreased diffusion of anti-cancer drugs through the spheroids and hypoxia could also contribute to drug resistance in 3D cultures^87^. Molecular studies are currently in progress to delineate the possible mechanisms for increased chemotherapeutics resistance in 3D cultures when compared to 2D cell monolayers.

## Conclusions

Based on the printability, shape fidelity, rheological parameters, scaffolds morphology, cell viability and rapid spheroid formation, Ink H4-RGD was found to be beneficial in developing tumor scaffolds with in-vivo stromal characteristics. This bioink can be crosslinked with cell culture medium, which facilitates good cell growth and scaffold stability. Ink H4-RGD printed NSCLC PDX cell laden scaffolds showed spheroid formation within 7 days. 3D printed spheroids showed more resistance to DOX, DTX and erlotinib in comparison to 2D monolayers of NSCLC PDX, MDA-MB-231 WT and HCC-827 cells.

## Methods

### Cell culture, hydrogels and reagents

NSCLC PDX tumor bearing mice [NOD.Cg-Prkdcscid Il2rgtm1Wjl/SzJ; TM00199 (LG0703F)] were received from Jackson labs, Bar Harbor, ME, USA. The tumors were then excised and grown in cultures using appropriate media conditions. Animals used in experiments were housed according to the regulations set by the American Association for Accreditation of Laboratory Animal Care under conditions of 37 °C and 60% humidity. All the experiments performed were priorly reviewed and approved by the Institutional Animal Use and Care Committee of Florida Agricultural and Mechanical University (protocol number: 018-04, Office of Laboratory Animal Welfare Assurance ID number: D16-00350 (A3581-01). MDA-MB-231 WT, A549, RT4, H460 and HCC-827 cells were also used for the study. Bioinks (Ink-H1, Ink-H2, Ink-H3, Ink H-4, Ink H4-RGD and Ink-H5) were received from TheWell Bioscience (North Brunswick, NJ 08902) company. These were formulated based on company’s tunable bioink system with different final concentrations such as Ink-H4 (VitroINK 3D) and Ink-H4-RGD (VitroINK RGD) of standard 1X concentration; Ink-H1, Ink-H2 and Ink-H3 of 0.2X, 0.5X and 2X concentration respectively and Ink-H5 of 0.75X concentration. The components of ink H4 and ink H4-RGD are proprietary information as they are being sold by TheWell Bioscience (North Brunswick, NJ 08902) company. To the best of our knowledge according to the information disclosed by The Well Bioscience (North Brunswick, NJ 08902) company, we only know that these inks simulate the natural extracellular matrix (ECM) environment by providing in-vitro anchorage and homing sites for in-vitro cells and spheroids. This polysaccharide-based hydrogel system (VitroGel) has also shielding and nurturing property, which closely mimics the in-vivo ECM. In comparison to the reported bioinks, Inks H4 and H4-RGD can be printed through extrusion due to their special shear-thinning and rapid recovery rheological properties of the hydrogel system and also can maintain the printed structure without further UV crosslinking. Moreover, these Inks H4 and H4-RGD can be mixed with different growth factors or compounds if needed to support different cell culture applications. DMEM/F12 media was purchased from Genesee Scientific (SanDiego, CA, USA). RPMI media, bFGF and EGF were purchased from Sigma-Aldrich (St. Louis, MO, USA). B27 supplement, NucBlue, Actin-Green reagents were purchased from Life technologies (USA). Live-Dead cell assay reagent was purchased from Biotium Inc (USA). E-cadherin and vimentin antibodies was purchased from Cell Signaling Technology (USA). Antirabbit, anti-mouse FITC and Rh conjugated secondary antibodies were purchased from Santa Cruz Biotechnology (USA). BIO-X and INKREDIBLE 3D bioprinter (CELLINK Inc, Sweden) were used to print all inks.

### Bioink optimization for scaffold fabrication

Ink H4 and H4-RGD were used for printability evaluation. Both these inks were prepared by our collaborator (The Well Bioscience Company). Based on the preliminary studies of VitroGel hydrogel systems, we observed that these formulated bioinks were found to be cross-linkable with cell culture medium. The experiment was designed to test printability of Ink H4 and H4-RGD, to determine crosslinker ratio (hydrogel:cell culture medium), crosslinking time, and also to evaluate printed scaffolds. For crosslinking and gelation study, each sample was diluted with a cell culture medium in the ratio (hydrogel to cell culture medium respectively) of 1:1, 1:2, 1:3, 1:4, 1:5, 2:1, 3:1, 4:1, 5:1, 6:1 and 10:1. For printability test, hydrogels were loaded into 3 mL printing cartridges and then connected to a controllable pressure regulator (0–1000 k pa) on a Bio-X printer. Three-layered (10 × 10 × 1 mm) scaffolds were printed on a petri-dish using 22G nozzle (410 µm diameter) and the printability was evaluated in terms of shape fidelity, applied extrusion pressure and post-printing cell viability. The line width and pore size of all the scaffolds were measured using Olympus IX71 microscope and cellSens Standard software (version 1.16; Tokyo, Japan).

The printability (Pr) of Ink H4 and Ink H4-RGD were defined based on the square shape using the following mathematical function

$${\text{Pr}} = \Pi {/4} \times {\text{1/C}} = {\text{L}}^{{2}} {\text{/16A}}$$

where A, C and L symbols indicate pore area, pore circularity and perimeter of the printed scaffold pore. Under ideal conditions, the printed lines of the constructs would be of square shape, and the Pr value was equal to 1. Larger Pr value indicates the greater gelation degree of the bioink and vice versa. For determining the Pr value of each print scaffold, perimeter and area of interconnected channels (n = 12), optical images of printed constructs were analyzed in Olympus IX71 microscope and cellSens Standard software (version 1.16; Tokyo, Japan).

### Cell printing and bioink cytocompatibility

Ink-H4 RGD was chosen for further biocompatibility evaluation studies. NSCLC-PDX, MDAMB-231 WT, A549, H460, HCC-827 and bladder cell (RT4) were used for biocompatibility evaluation of Ink H4 RGD. For each cell line, 400 µL of cell suspension (5 × 10^6^ cells) was mixed homogenously in 2 mL of H4-RGD bioink by a sterile spatula and loaded carefully into the printing cartridge. Conical shape 22G nozzle was used for printing all cell lines throughout the biocompatibility experiments. The final printed cell laden scaffolds had 10 × 10 × 1 mm dimensions with an estimation of 50,000 cells per each scaffold. All printing procedures were carried out at room temperature in 12 well plates and each cell-laden scaffold was covered with 500 µL organoid culture medium and then incubated at 37 °C and 5% CO_2_. Organoid cell culture medium was prepared from DMEM F-12/and RPMI with EGF (Epidermal growth factor), bFGF (basic fibroblast growth factor) and B-27 complement. The cell viability was evaluated before printing (simple gel cell mix) and after printing on day 1(immediately), 2, 4, 7 and 10 by using a live and dead cell assay kit. Briefly, printed cells were stained with 2 µM calcein-AM/4 µM EthD-III for 1 h and washed twice with serum free media. Thereafter, the images were acquired and analysed by using Olympus IX71 microscope and cellSens Standard software (version 1.16; Tokyo, Japan) respectively. Relative percentage of live and dead cells were determined by counting the cells using NIH ImageJ software (1.43u; https://imagej.nih.gov/ij).

### Rheological properties of Ink H4 and H4-RGD

Malvern Kinexus Pro + dynamic rheometer (Malvern Panalytical, UK) with a 20 mm parallel plate of rough surface geometry was used for the evaluation of rheological properties of cell free hydrogels. The viscoelasticity and viscosity of the bionks were evaluated with oscillation-time sweep, flow-sweep, oscillation-temperature ramp and repeated amplitude sweep procedures. Briefly for rheology studies, samples were prepared by two methods, which includes (1) hydrogel without crosslinker (i.e., without cell culture medium) and (2) hydrogel with crosslinker (i.e., with DMEM-F12 medium) at 5:1 ratio. For each measurement, about 200 µL sample was used with a measuring gap of 0.5 mm and the edge of the sample was covered with ultra-low viscosity silicone fluid (viscosity of 5cSt at 25 °C, Clearco, USA) to prevent sample drying. For samples with cell culture media, 200 µL hydrogel (H4 and H4-RGD) was mixed with 40 µL of DMEM-F12 medium. The viscosity was evaluated at shear rates ranging from 0.1 to 100/S at 25 °C. The oscillation temperature ramp evaluation was carried out from 20 to 40 °C with 2 °C/min ramp and 1 Hz.

The shear thinning and recovery properties of the hydrogel were evaluated with repeated time-sweep and amplitude-sweep testing procedures at 25 °C and 37 °C. Briefly, the hydrogel sample was loaded on the bottom plate of the rheometer and tested under 0.1% shear strain for 10 min. First testing stage revealed the stability of the hydrogel and its ability to extrude easily. After first stage, an amplitude sweep was applied by increasing the shear strain from 0.1 to 500% within 4 min. This second stage mimicked the extrusion process, which makes hydrogel flow due to decrease in the elastic modulus when the shear strain was increased. Right after the amplitude sweep, the second time sweep test was immediately applied to evaluate the rapid recovery of the elastic modulus of the hydrogel after printing. The shear stress vs shear rate graph was represented on log–log plot and then the intercept and R^2^ values were determined. The following adjustable parameters and terms; τ (shear stress), γ (shear rate), K (consistency index; gives an idea of the viscosity) and n (flow behaviour index; dimension less) were analysed by simple power law model i.e., τ = kγ^n^, where n < 1 denotes pseudoplastic and n > 1 indicates dilatants flow.

To test the rheology of fully crosslinked hydrogel, about 1.2 mL hydrogel from either non crosslinked or partial crosslinked hydrogels (5:1 mix) was added to a 35 mm petri dish and then covered with 2 mL DMEM-F12 medium. The samples were incubated for 24 h and the rheology was evaluated with oscillation-time sweep procedure at 37 °C.

### Stiffness and stability of printed cell laden scaffolds

The rheology of cell laden scaffolds was assessed using AR 1500Ex Rheometer (TA instrument, USA) with parallel-plate geometry (20 mm, taper angle of a = 0.0412 and rad = 2.36). Briefly, cells were printed with designed scaffolds of 20 mm diameter, 1 mm height and 45% infill density and were incubated in six well plates supplemented with DMEM-F12 organoid media at 37 °C and 5% CO_2_. Oscillation time sweep with a strain of 1% and angular frequency of 10 rad/s was used for cell laden stiffness evaluation and the stiffness was measured during day 1, 5, 10 and 15. Each cell laden scaffold was constructed from Ink H4-RGD with NSCLC-PDX cells (2 × 10^5^ cells per scaffold).

### Characterisation of 3D spheroids by using NucBlue and actin green assay

Nucleus and Actin staining (NucBlue and Actin green assay) were performed to confirm the spheroids formation and their growth in the printed scaffolds. The media was removed from the scaffolds and then the scaffolds were subjected to fixation by using 4% formaldehyde for 1 h at room temperature and washed with 1 mL of HBSS (twice). The scaffolds were then permeabilized with 0.1% Triton X-100 and subsequently after washing with HBSS, staining with NucBlue and ActinGreen488 (Life Technologies) was performed according to the manufacturer’s protocol. The assay was performed after 48 h, 72 h, on 5th day and 7th day. Images were then acquired by using Olympus IX71 microscope and cellSens Standard software (version 1.16; Tokyo, Japan).

### Scanning electron microscopy (SEM) analysis of the spheroid structure

NSCLC-PDX spheroids (on the 10th day) were fixed with 4% glutaraldehyde for 30 min. Fixed spheroids were subsequently dehydrated with graded proportions of ethanol (25, 50, 75, 95, and 100%). Afterwards, complete dried scaffolds were coated with palladium gold, and finally examined under a scanning electron microscope (JEOL) at an acceleration voltage of 5 kV.

### Immunofluorescence staining of 3D spheroids for assessment of in vitro tumor microenvironment, which mimics the in vivo stromal characteristics

On the 7th day, printed cell-laden scaffolds (i.e., NSCLC PDX spheroids) were fixed with 4% formaldehyde for 30 min at 37 °C, washed thrice with PBST and permeabilized with 0.4% Triton for 30 min. The triton washed out and blocked with 5% BSA overnight, washed thrice with PBST and incubated with E-Cadherin-rabbit and Vimentin-rabbit overnight. Then the scaffolds were washed thrice with PBST and further stained with the secondary antibodies for 3 h. Finally, the scaffolds were washed thrice with PBST and stained with NucBlue for 30 min. Images were taken with Olympus IX71 microscope.

### Cytotoxicity assays

For cytotoxicity study, 4 mL of Ink H4-RGD was mixed uniformly with 800 µL cell suspension (containing 1.2 × 106 cells), loaded into the printing cartridge and then printed onto the 24 well plates with estimated cell density of 5 × 10^4^ cells/scaffold. Each printed cell-laden scaffold was nourished with 250 µL of organoid medium every other day and incubated at 5% CO_2_ and 37 °C. On day 5, the cell-laden scaffolds (with MDAMB-231 WT, NSCLC-PDX and HCC 827 cells) were treated with anticancer drugs erlotinib, DTX and DOX. DOX was not usually used against lung cancer and hence was not used for NSCLC-PDX). After 48 h incubation, the scaffolds were stained with 300 µL of MTT (0.05 mg/mL) for 4 h and the formazan crystals were dissolved with 250 µL of DMSO for 2 h. For comparison, conventional 2D cell culture models with equal cell density were also prepared for each cell line. Briefly, 5 × 10^4^ cells were plated in 24 well plates and incubated at 37 °C and 5% CO_2_ for 48 h. After 48 h, all the cells were treated with similar drugs (Erlotinib, DTX and DOX) separately. Then, MTT solution (0.05 mg/mL) was added for 3–4 h. Finally, the formazan crystals were dissolved with 250 µL of DMSO for 30 min. The absorbance was measured at a wavelength of 565 nm on a Fluoroskan Ascent FLTM instrument (Labsystem). Cytotoxic activity was expressed as the drug concentration that inhibited cell viability by 50% (IC_50_).

### Statistical analysis

The experimental results were expressed as mean ± standard deviation. Statistical analyses between different groups were performed by one-way ANOVA. p value < 0.05 was considered as statistically significant.

## Supplementary Information

Supplementary Figures.

## Data Availability

All the data generated or analyzed during this study are included in this published article (and its “Supplementary Information” files).
