# Supplementary material for: Polysaccharide hydrogel based 3D printed tumor models for chemotherapeutic drug screening
Source: Sci Rep. 2021 Jan 11;11:372. doi: 10.1038/s41598-020-79325-8 (PMC7801509; doi:10.1038/s41598-020-79325-8)
Supplement: Supplementary file 1 — Supplementary Figures. [file 41598_2020_79325_MOESM1_ESM.docx]

**Polysaccharide hydrogel based 3D printed tumor models for chemotherapeutic drug screening**

Aragaw Gebeyehu^#1^, Sunil Kumar Surapaneni^#1^, John Huang^2^, Arindam Mondal^1^, Vivian Ziwen Wang^2^, Nana Fatima Haruna^2^, Arvind Bagde^1^, Peggy Arthur^1^, Shallu Kutlehria^1^, Nil Patel^1^, Arun Rishi^3^ and Mandip Singh^1^*

^#^Aragaw Gebeyehu and ^#^Sunil Kumar Surapaneni contributed equally to this work and listed as co-first authors.

^1^College of Pharmacy and Pharmaceutical Sciences, Florida A&M University, Tallahassee,

FL32307, USA

^2^TheWell Bioscience, North Brunswick, New Jersey, 08902, USA

^3^John D. Dingell VA Medical Center, Detroit, MI, 48201, USA; Department of Oncology,

Karmanos Cancer Institute, Wayne State University School of Medicine, Detroit, MI, 48201, USA

*Corresponding Author:

Prof. Mandip Singh, Ph.D.

College of Pharmacy and Pharmaceutical Sciences, Florida A&M University, Tallahassee, Florida, 32307, USA.

Tel: +1- 850-561-2790 Fax: 850-599-3813; E-mail: mandip.sachdeva@gmail.com

**Figure S1:** Bioprintability of NSCLC-PDX cell laden scaffolds with Inks H1, H2, H3 and H5

**B)**

**
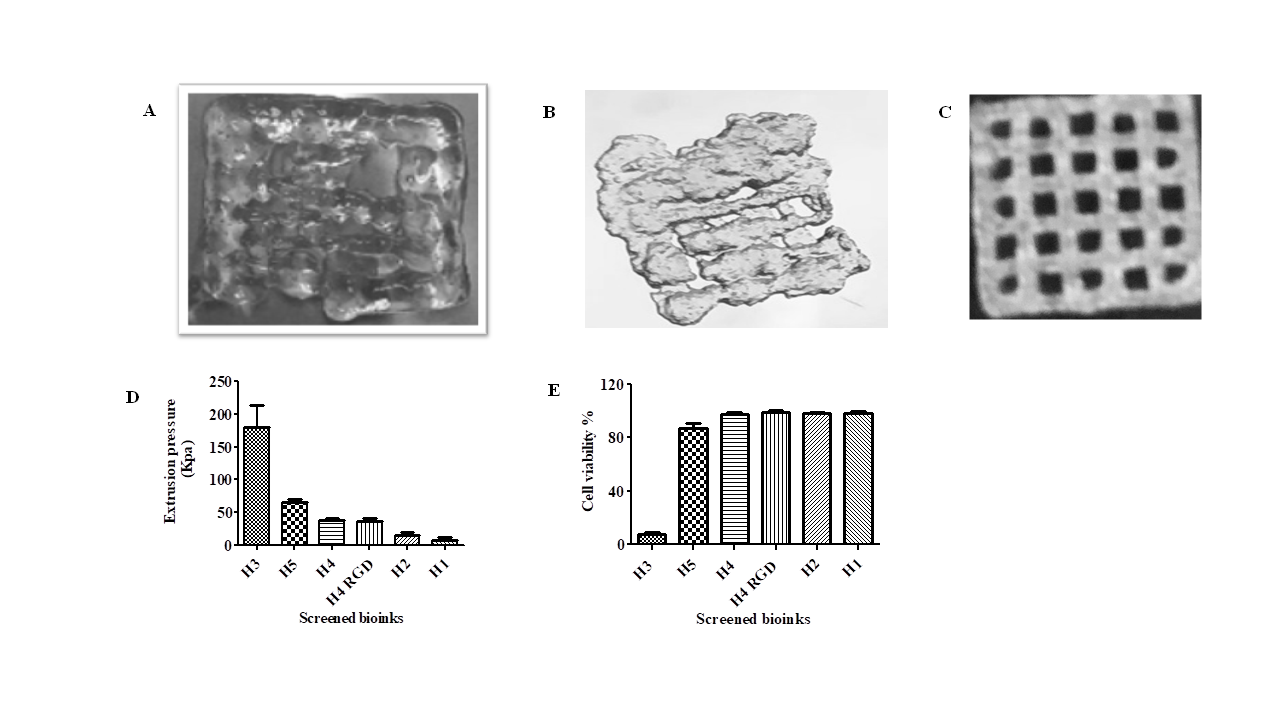

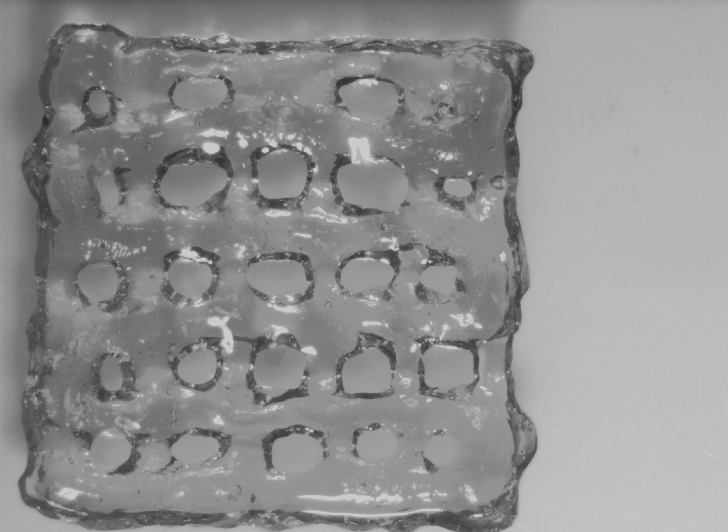
**

**A)**

**Ink H1**

**Ink H2**

**D)**

**C)**

**Ink H3**

**Ink H5**

**
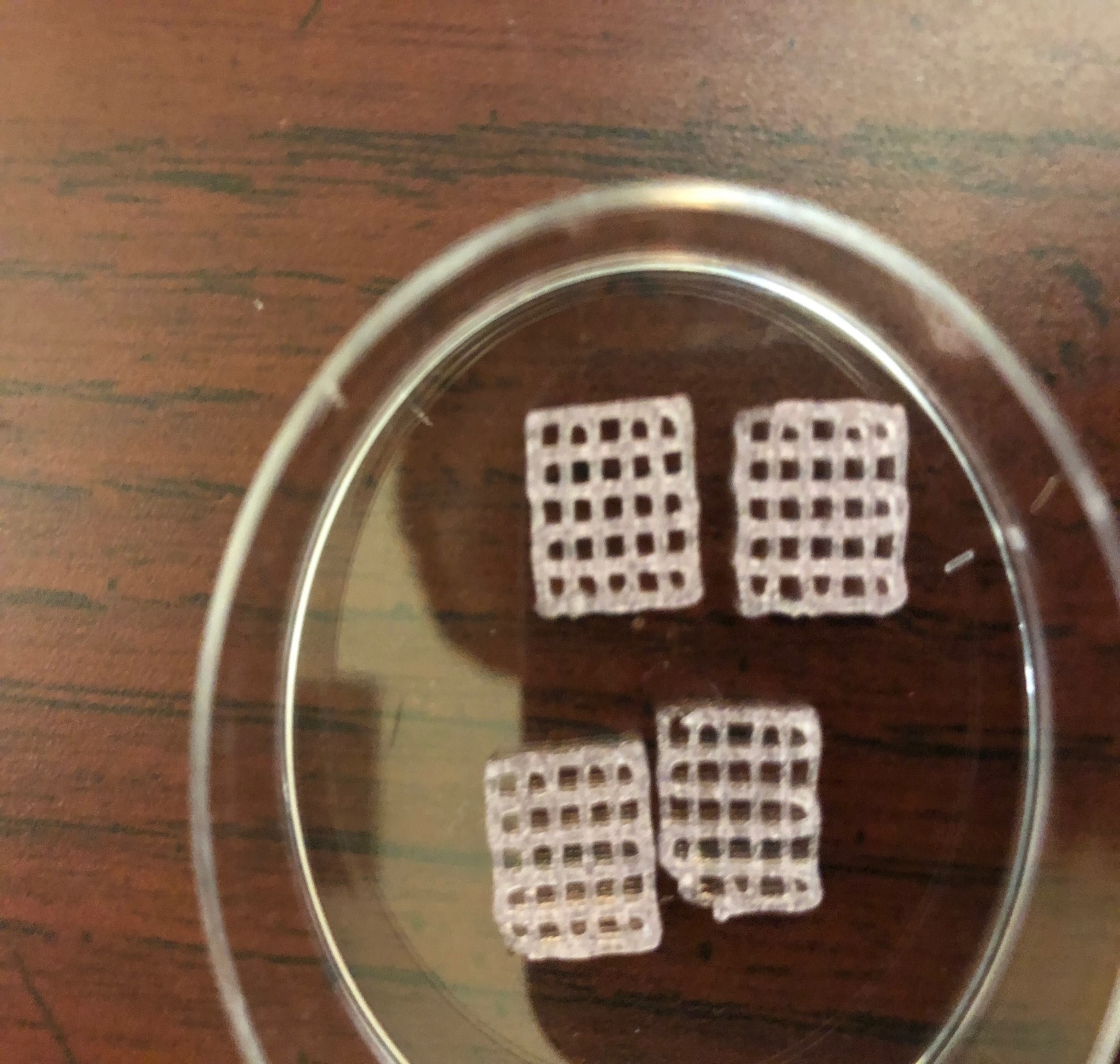

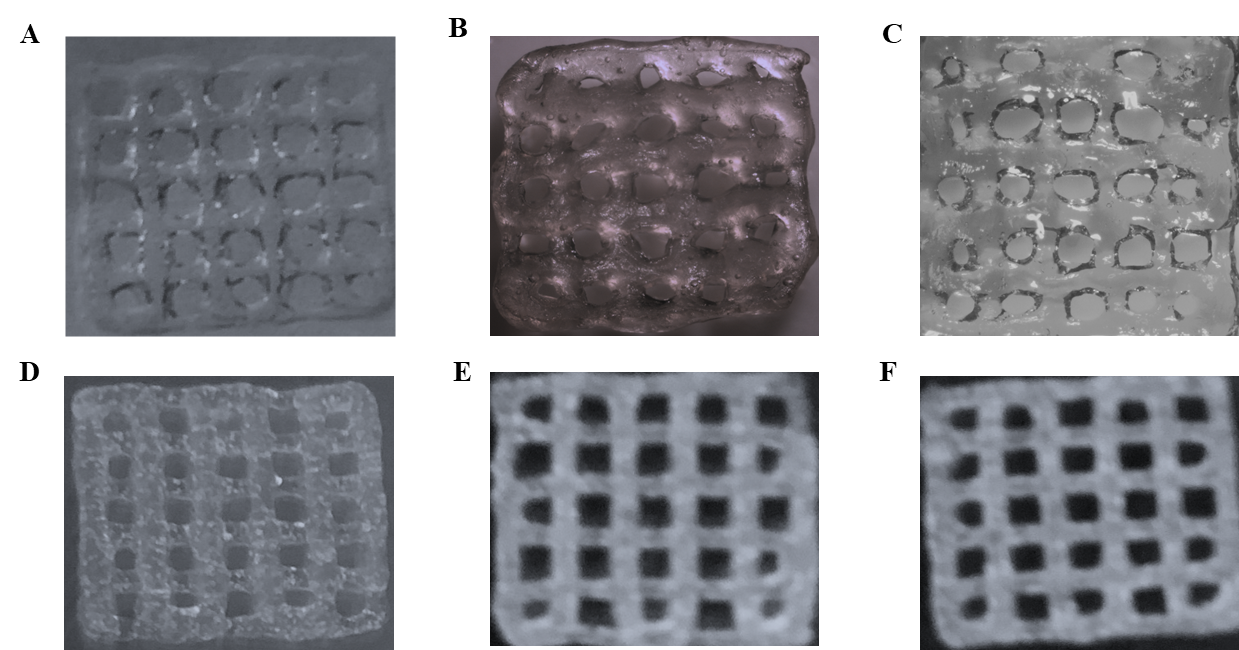
**

**Figure S2:** Cell viability of NSCLC-PDX cell laden scaffolds printed with Inks H3, H5, H2 and H1

**
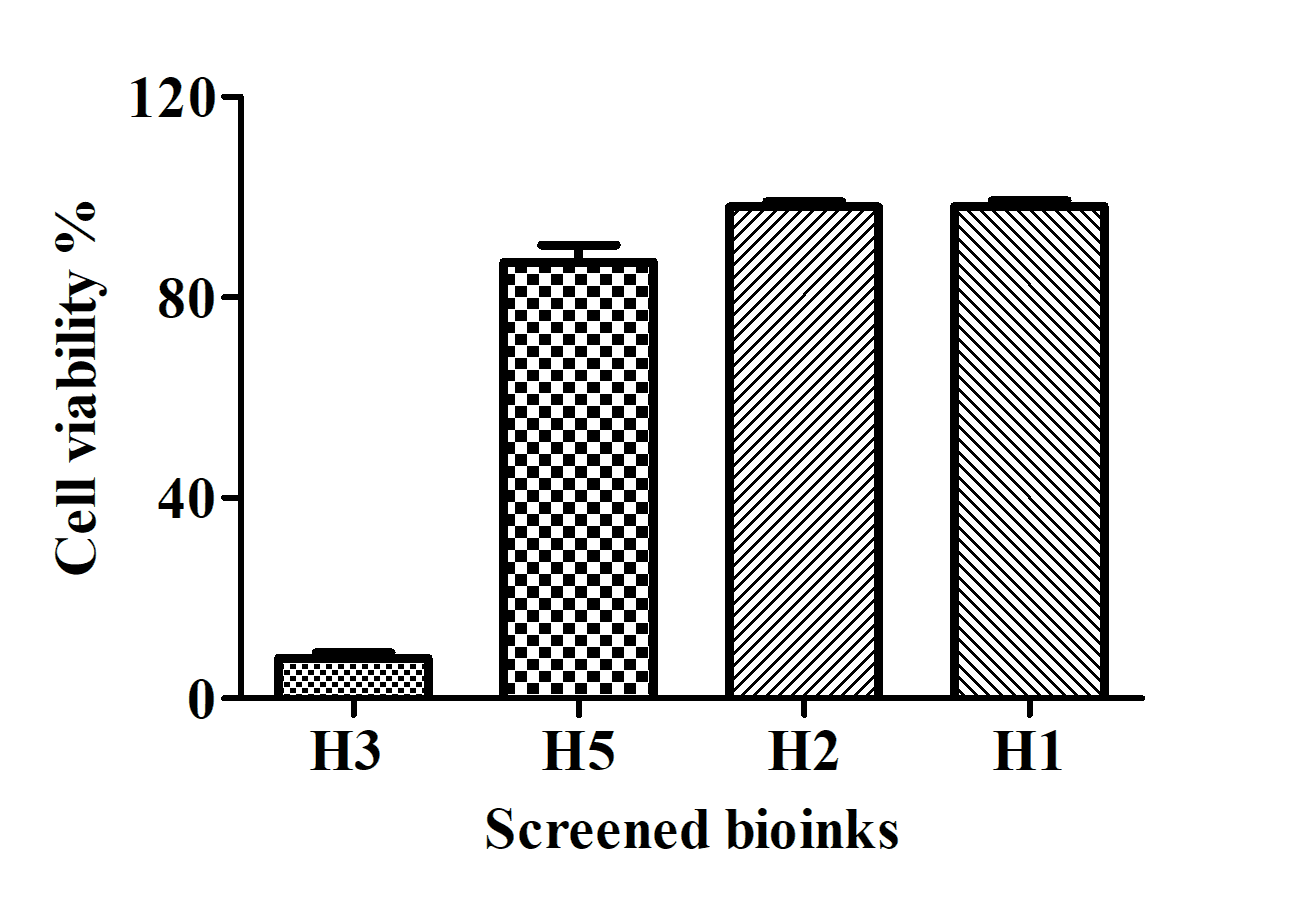
**

**Figure S3:** Extrusion pressure required for printability of NSCLC-PDX cells with Inks H3, H5, H2 and H1

**
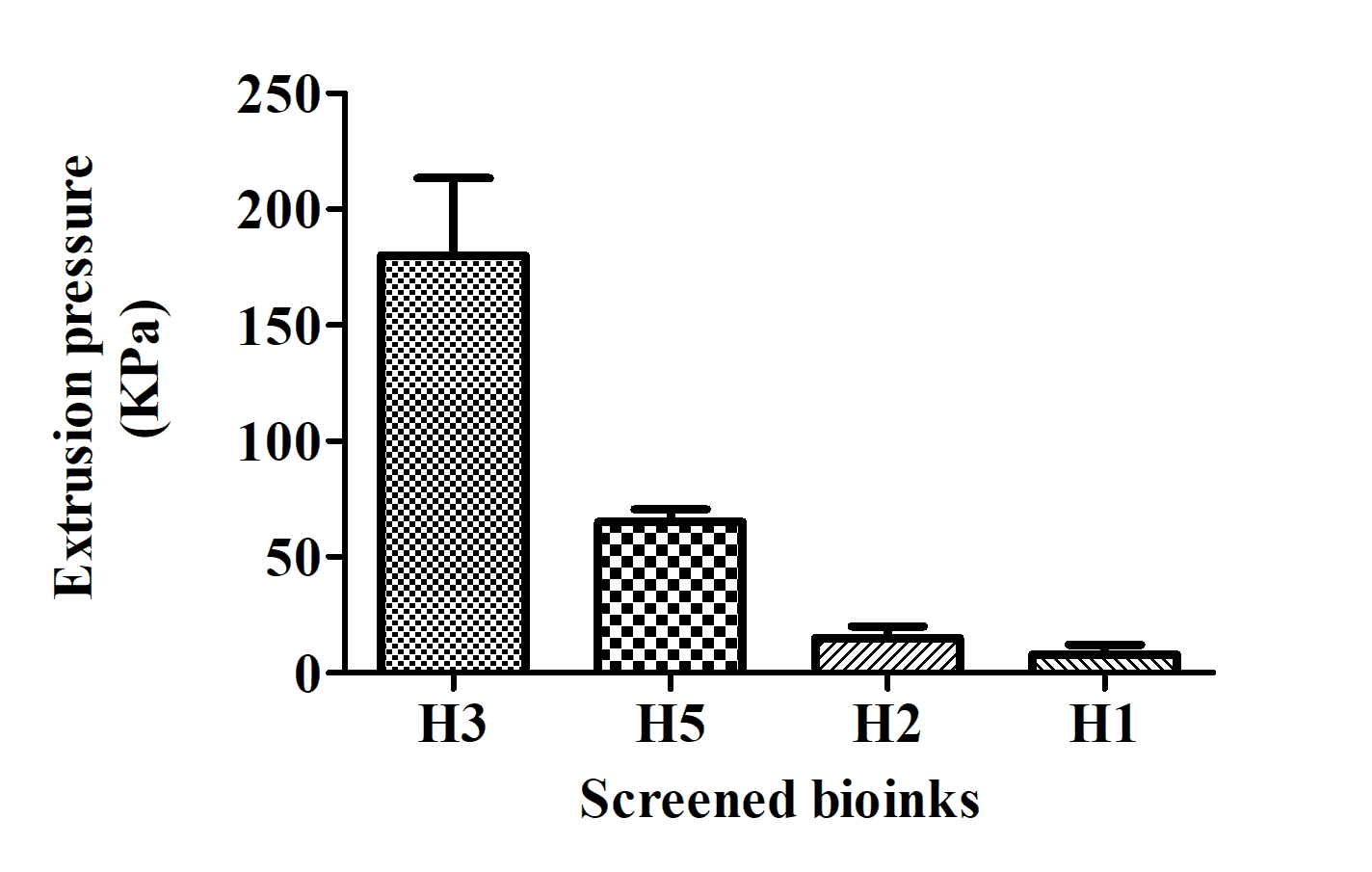
**
